# Zinc binding groups for histone deacetylase inhibitors

**DOI:** 10.1080/14756366.2017.1417274

**Published:** 2018-04-04

**Authors:** Lei Zhang, Jian Zhang, Qixiao Jiang, Li Zhang, Weiguo Song

**Affiliations:** a Department of Medicinal Chemistry, School of Pharmacy, Weifang Medical University, Weifang, Shandong, China;; b School of Pharmacy, Qingdao University, Qingdao, Shandong, China

**Keywords:** Histone deacetylase inhibitor, zinc binding group, anti-tumour, selectivity, drug design

## Abstract

Zinc binding groups (ZBGs) play a crucial role in targeting histone deacetylase inhibitors (HDACIs) to the active site of histone deacetylases (HDACs), thus determining the potency of HDACIs. Due to the high affinity to the zinc ion, hydroxamic acid is the most commonly used ZBG in the structure of HDACs. An alternative ZBG is benzamide group, which features excellent inhibitory selectivity for class I HDACs. Various ZBGs have been designed and tested to improve the activity and selectivity of HDACIs, and to overcome the pharmacokinetic limitations of current HDACIs. Herein, different kinds of ZBGs are reviewed and their features have been discussed for further design of HDACIs.

## Introduction

Histone deacetylases are a family of enzymes that are responsible for removing acetyl groups from the ɛ-*N*-acetyl group of histone lysine residues^1–3^. A total of 18 different isoforms of HDACs (which are classified into four classes) have been discovered. Class I (HDAC1, 2, 3, and 8), II (HDAC4–7, 9, and 10), and IV (HDAC11). HDACs are zinc-dependent enzymes which require the zinc ion for the catalytic reaction. On the other hand, class III HDACs (Sirt1–7) are a group of nicotinamide adenine dinucleotide (NAD^+^)-dependent enzymes. Enzymes in class I and II are known as classical HDACs, which attracted enormous attentions in the HDAC function exploration and inhibitor development.

**Figure 1.**
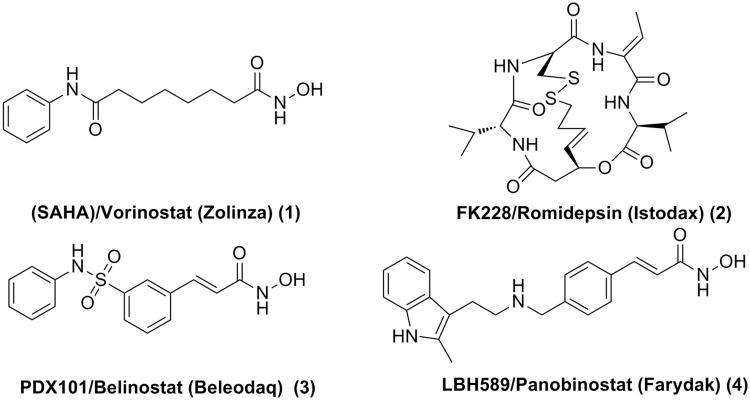
Structures of HDACIs approved by the US FDA.

Overexpression and aberrant recruitment of HDACs play crucial roles in a variety of diseases such as neurodegenerative diseases^4^^,^^5^, infection^6^^,^^7^, human immunodeficiency virus (HIV)^8–10^, cardiac diseases^11^^,^^12^, and especially tumour^13^. Inhibitors of HDACs have received considerable attention, in particular due to their potential as antitumor agents. Four HDAC inhibitors (HDACIs) have been approved by US Food and Drug Administration (FDA) for the treatment of cancer (Figure 1). Suberoylanilide hydroxamic acid (SAHA)/Vorinostat (Zolinza) was approved in 2006 for the treatment of refractory and relapsed Cutaneous T-cell lymphoma (CTCL)^14^. The cyclic tetrapeptide HDACI, FK228/Romidepsin (Istodax)^15^, was also approved for treatment of CTCL. PDX101/Belinostat (Beleodaq)^16^ and LBH589/Panobinostat (Farydak)^17^ were approved for the treatment of peripheral T-cell lymphoma (PTCL) and multiple myeloma, respectively (Figure 1).

**Figure 2.**
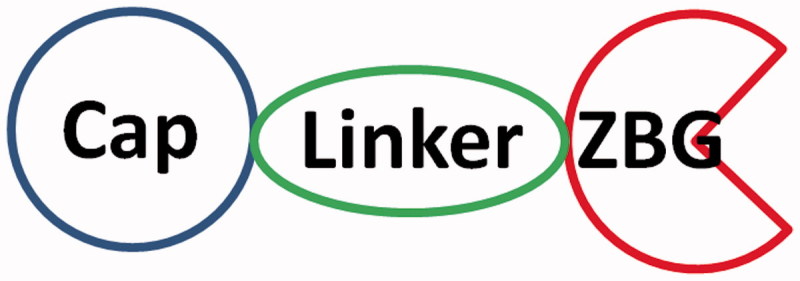
Pharmacophore of HDACIs.

Structures of HDACIs are generally characterised by a zinc binding group (ZBG), a cap, and a linker that combines the above two parts together (Figure 2). The cap and linker bind to residues in the active site of HDACs, thus contribute to the ligand–receptor interactions and affect the selectivity of HDACIs. Meanwhile, binding of ZBGs to the zinc ion and surrounding residues play the decisive role in the inhibitory activity of HDACIs^18^. Although different ZBGs have been evaluated and reported, modifications in the cap and linker still received more attention in the development of potent and selective HDACIs^19^. Structural modification in the ZBGs of current HDACIs might be a more efficient way of getting HDACIs to the market. In consideration of the importance of ZBGs as functional moieties in the structure of HDACIs, different kinds of ZBGs as well as their advantages and disadvantages are systematically reviewed in the present work.

## Classic ZBGs

The commonly used ZBGs, such as hydroxamic acid and benzamide, along with other ZBGs from HDACIs that had been approved by FDA or currently being investigated in clinical trials (such as carboxylic acid and thiol), are classified as classic ZBGs. These ZBGs have been widely accepted in the development of HDACIs, and their characteristics have been extensively studied^20^. The classic ZBGs are characterised by high activity, selectivity, but also off-target effects, potential toxicities and instability *in vivo*^21^.

### Hydroxamic acid

**Figure 3.**
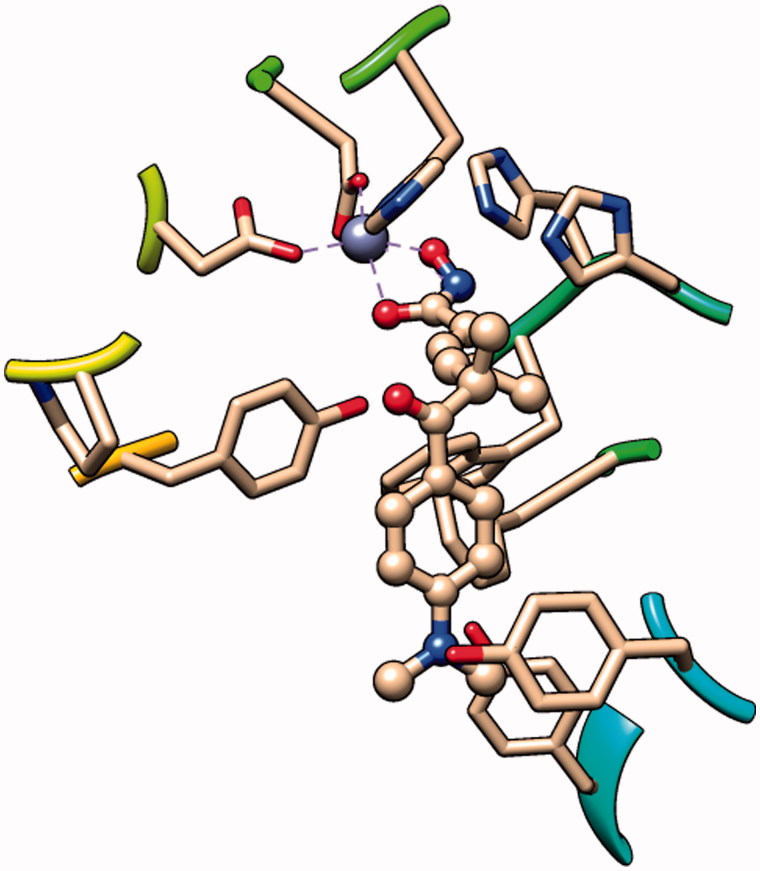
The binding pattern of TSA in the active site of HDAC8 (PDB entry: 1T64).

Hydroxamic acid is the most commonly used ZBG for its strong binding affinity with the zinc ion. By analysis of the crystal structure of the inhibitor–receptor complex, it was elucidated that hydroxamic acid binds to the zinc ion in a chelating manner (Figure 3), which is considered as a guarantee of inhibitor activity. The naturally derived compound Trichostatin A (TSA), which showed very high HDAC inhibitory and antiproliferative activities, was equipped with hydroxamic acid as ZBG^22^. The first HDAC inhibitor approved by the FDA, SAHA, also utilised the hydroxamic acid as the ZBG. Moreover, most HDACIs currently in clinical trials shared structural similarities with TSA and SAHA, using the hydroxamic acid as ZBGs^23^.

The advantages of hydroxamic acid group as ZBG include superior zinc binding ability, fair *in vitro* stability, good solubility, and easy synthesis. Therefore, in most cases, this group was the preferred ZBG in the design of new HDACIs. Various groups have been designed to mimic the hydroxamic acid group, but few exhibited higher potency^24^. The disadvantages of hydroxamic acid group as ZBG in HDACIs are also obvious. The selectivity of hydroxamic acid group is often questioned in the drug design, because of its high binding affinity to the zinc and other ions^25^. Undesirable side effects can also result from the binding of hydroxamic acid group to other zinc-dependent enzymes such as aminopeptidases, matrix metalloproteinases, and carbonic anhydrase. The common approaches to improve the selectivity of HDACIs with hydroxamic acid group are structural modifications in the linker and cap regions. Poor pharmacokinetics (rapid degradation and clearance *in vivo*) were also associated with HDACIs with hydroxamic acid as ZBG^26^.

### Benzamide

**Figure 4.**
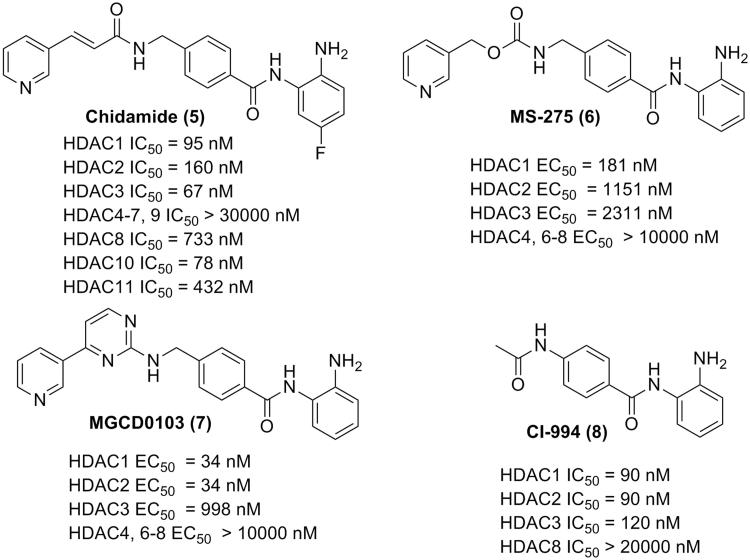
Structures of the benzamide HDACIs.

Benzamide derivatives are a big class of HDACIs with superior class I selectivity compared with hydroxamic acid HDACIs. The crystal structure analysis revealed chelation of the zinc ion by the amino group in the benzamide. Additionally, the distance between the carbonyl oxygen and zinc is more than 2.5 Å, indicating weak interactions. Chidamide (Epidaza)^27^ is a benzamide HDACI approved by Chinese Food and Drug Administration (CFDA) for the treatment of relapsed or refractory PTCL (Figure 4). MS-275^28–31^ (HDAC1 selective) and MGCD0103^32–36^ (HDAC1, 2 selective) are two benzamide HDACIs designed for the treatment of both hematologic malignancies and solid cancers, which are currently in clinical trial as mono therapies or in combination with other drugs. Good performances in clinical trials were also observed for another benzamide derivative, CI994^37–40^.

The most significant feature of benzamide as ZBG in HDACIs is class I selectivity or individual HDAC isoform selectivity. Side effects of HDACIs are supposed to be reduced with the improvement of selectivity. The highly selective HDACIs could also serve as probe molecules in the diagnosis and pathogenetic research of diseases involving only a specific isoform of HDACs. The major disadvantage of benzamide derivatives is relatively compromised therapeutic benefits in clinical trials, which might explain the fact that, none of the benzamide HDACIs has been approved by the US FDA yet. Although high selectivity implies safety in clinical application, the exposed amino group in the benzamide structure could potentially induce *in vivo* toxicity^41^. Furthermore, tumour cells are more likely to develop drug resistance against highly selective inhibitors, which might compromise the therapeutic effects of benzamide HDACIs in long-term therapies^42^.

### Carboxylic acid

**Figure 5.**
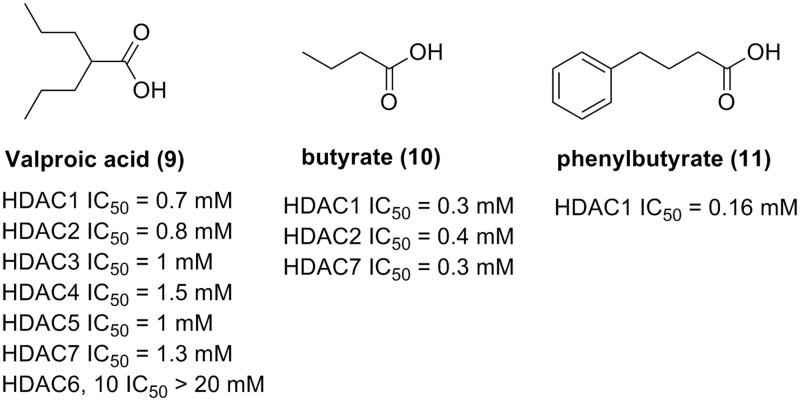
Carboxylic HDACIs in clinical trials.

Carboxylic acid derivatives are a small group of HDACIs. Due to their weak zinc ion binding abilities, carboxylic acid HDACIs have not attracted as much attention as HDACIs with hydroxamic acid or benzamide ZBGs. The short chain fatty acids in clinical trials, such as valproic acid^43^^,^^44^, butyric acid^45^, and phenylbutyrate^46^, only exhibited IC_50_ in the magnitude of mM, as revealed by the *in vitro* enzymatic inhibitory assay (Figure 5). Nonetheless, the marketing potential of compounds in this group is still under evaluation. Current activity data derived from the clinical trials indicated the potential of application of these fatty acids in tumour treatment.

### Thiols

Thiol group is commonly used for metal binding in the drug design, with the most famous example of Captopril. The first natural HDACI approved by US FDA, FK228, was regarded as a pro-drug, which can be metabolised to its active form via glutathione conjugation. The thiol group exposed in this process was supposed to chelate the zinc ion. Many thiol derivatives have been reported by structural modification of SAHA^47^ and potent natural products^48^ or by *de novo* design^49–53^. However, none of them has been gained access to the clinical research so far.

Since sulfydryl group is a strong ZBG which makes few contributions to the selectivity of HDACIs, the selectivity can be adjusted by structural modifications in the linkers and caps of HDACIs. Introduction of thiol group to HDACIs also increases the risk of side effects caused by binding to other metal-dependent targets *in vivo*.

## Novel ZBGs

Since classic ZBGs possess both obvious advantages and disadvantages, efforts have been made in discovery of more ideal ZBGs for HDACIs. Classes of HDACIs with novel ZBGs featured with high selectivity, potency, and stability have been designed and synthesised^19^. Although none of the HDACIs with novel ZBG has gained access to clinical trial currently, such investigations guaranteed the rise of new generations of HDACIs with improved pharmacological and pharmacokinetic profiles.

**Figure 6.**
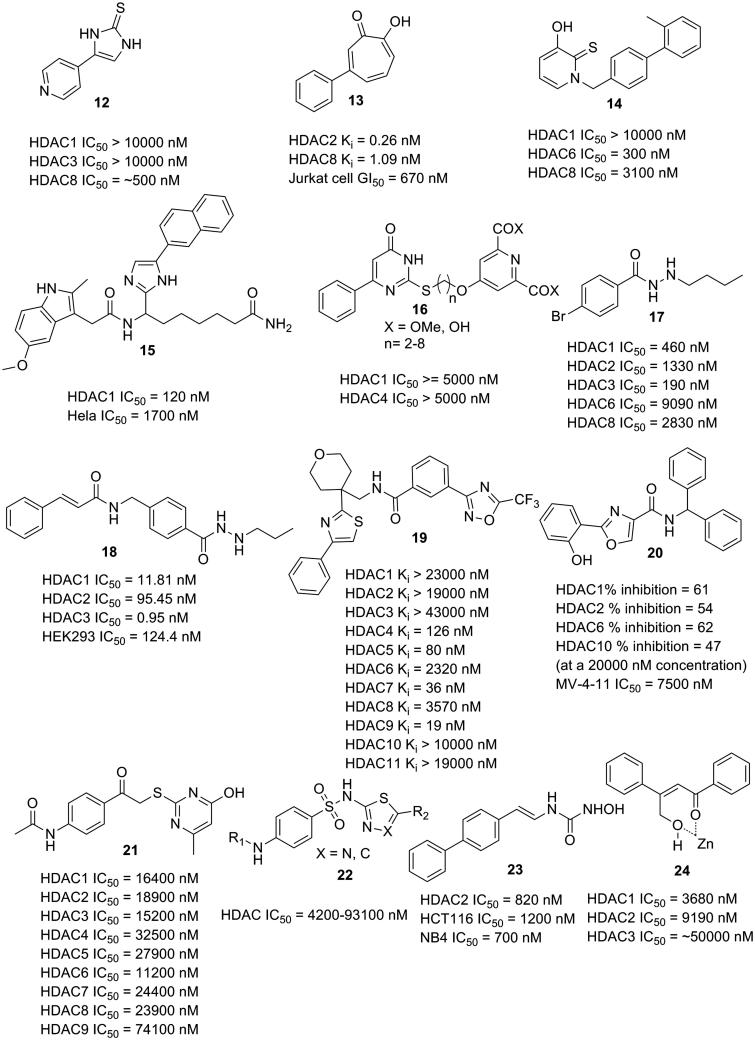
HDACIs with novel ZBGs.

An imidazole thione containing molecule (**12**), which showed HDAC8 selectivity over HDAC1 and HDAC3, was discovered by an enzyme based screening approach (Figure 6)^54^. The imidazole thione group supposedly could bind to the zinc ion, thus could serve as a candidate ZBG for the further design of selective HDACIs. Molecules with pyrimido[1,2-c][1,3]benzothiazin-6-imine scaffold were also discovered to selectively target HDAC8^55^.

A series of tropolone derivatives were reported by Wright and co-workers as HDAC2 selective inhibitors^56^. Molecule **13** displayed high levels of selectivity for HDAC2 (*K_i_* = 0.06 nM) comparing with HDAC1 (not active), HDAC4 (*K_i_* = 10860 nM), HDAC5 (not active), HDAC6 (not active), and HDAC8 (*K_i_* = 1.47 nM). It can also potently inhibit the growth of several tumour cell lines in the antiproliferative assay. In the molecular docking analysis, simulation results revealed that the tropolone group is capable of zinc ion binding. These investigations into tropolone-based HDACIs could give rise to a new chemotype of HDACIs with high potency and selectivity.

Oyelere and co-workers identified 3-hydroxypyridin-2-thione (3-HPT) as a novel ZBG, and the 3-HPT derivatives were HDAC6 selective inhibitors with no inhibition of HDAC1^57^. In this series, molecule **14** exhibited HDAC6 selectivity with inhibitory IC_50_ value of 300 nM compared with HDAC8 (IC_50_ value of 3100 nM). Docking studies revealed that the 3-HPT group functions as ZBG. It is valuable for the 3-HPT derivatives in the development of specifically selective HDACIs for the treatment of tumour and other diseases without interference with HDAC1.

Several hydroxamic acid substitutes have been designed and synthesised as ZBGs by Attenni and co-workers, and the inhibitory activities were evaluated^58^. Primary amide containing molecules exhibited good performance in the enzymatic inhibition and antitumor assays. Molecule **15** with primary carboxamide moiety functioned as ZBG showed selective inhibitory pattern for HDAC1, and demonstrated antitumor efficacy in a xenograft model comparable to vorinostat. These findings revealed that primary carboxamide group could be used as an alternative ZBG for further HDACI design.

Chelidamic derivatives have been reported as novel HDACIs by Mai and co-workers^59^. Molecules with chelidamic scaffold represented as the zinc binding group (**16**) exhibited inhibitory effects on both HDAC1 and HDAC4. Multiple analyses, including cell cycle analysis, apoptosis induction, and granulocytic differentiation analysis, have revealed that the representative molecules have the potential to serve as leading compounds in the cancer treatment. Chelidamic group as ZBG is considered as a promising candidate in the development of selective HDACIs by further structural modification.

Liao and co-workers discovered a class of HDACIs with benzoylhydrazide scaffold as the ZBGs^60^. Molecule **17** exhibited class I selectivity, especially effective against HDAC3 (IC_50_ value of 0.06 μM). Subsequent *in vitro* assays with molecule **17** revealed potent anti-proliferative activities along with less overt cytotoxicity comparing with both SAHA and MS-275. Other *in vitro* evaluations emphasised the potential of benzoylhydrazide as new chemotype of HDACIs as well. Recently, Chou and co-workers also reported a series of hydrazide derivatives as potent class I HDACIs^61^. The SAR (structure–activity relationship) analysis revealed that a 3-carbon-length β-nitrogen alkyl substituent chain provides ideal activity. One of the most potent molecule, **18**, exhibited HDAC3 inhibitory activity of 0.95 nM (IC_50_ value), and EC_50_ values of 36.37, 76.64, and 151.7 nM against MV4–11, Molm14, and RS4–11 cell lines, respectively.

HDACIs with hydrazide motif are of significant importance as a new generation. Without the dependence on the zinc binding interactions, these molecules are supposed to exert less off-target effects. Moreover, the reported hydrazide derivatives showed class I selectivity (especially HDAC3), indicating the potential of successful disease treatment without serious adverse reactions. However, long-term use safety is yet to be confirmed for those HDACIs with hydrazide group.

A distinct class of HDACIs using trifluoromethyloxadiazolyl (TFMO) moiety as ZBG was reported by Nolan and co-workers^62^. Binding patterns of these molecules were elucidated to be a non-chelating manner by crystallographic approaches. Zinc binding of TFMO derivatives is mediated by a fluorine atom in the trifluoromethyl group and aided by an oxygen atom in the oxadiazole heterocycle. In the enzymatic inhibition assay, these TFMO containing HDACIs exhibited high selectivity for HDAC class IIa. Molecule **19** displayed *K_i_* values of 0.126, 0.080, 0.036, and 0.019 μM against HDAC4, 5, 7, and 9, respectively. In contrast, the *K_i_* values of molecule **19** against HDAC6, HDAC8, and the rest HDACs (HDAC1, 2, 3, 10, 11) are 2.32, 3.57, and >10 μM, respectively. These class IIa selective HDACIs were further utilised to reveal the gene regulation mechanisms of class IIa HDACs. The discovery of TFMO-based HDACIs supports the design of selective HDACIs being of importance in treatment of a specific disease, and exploration of underlying mechanisms as probe molecules.

Woster and co-workers reported the 2-(oxazol-2-yl)phenol moiety as a novel ZBG that can be used for the design of potent HDACIs^63^. A series of 2-(oxazol-2-yl)phenol derivatives were synthesised and tested in the activity assay. The derived molecules exhibited potent HDAC1, HDAC6 and HDAC10 inhibitory activities. Among these compounds, molecule **20** (IC_50_ 7.5 μM against MV-4–11 leukemia cell line) could efficiently induce the acetylation of histone 3 lysine 9 (H3K9) and p21^Waf1/CIP1^ compared with SAHA. Molecular modelling analysis revealed that 2-(oxazol-2-yl)phenol group shows a similar zinc-binding pattern as the benzamide group in the ligand of a crystal structure (PDB entry: 4LY1). Although no remarkable selectivity was observed, the 2-(oxazol-2-yl)phenol derivatives are considered promising candidates as ZBG in the development of novel and potent HDAC inhibitory drugs.

Hydroxypyrimidines (**21**) without contribution to the selectivity of HDACs were discovered to be a new ZBG^64^. The hydroxyl group and the pyrimidine group in the ZBG are both critical for activity, as revealed by SAR studies. Park and co-workers have identified *N*-[1,3,4]thiadiazol-2-yl and N-thiazol-2-yl sulfonamide groups as promising ZBG (**21**) with probably better physicochemical properties than hydroxamic acids, as demonstrated by the clinical studies^65^. The oxygen atoms of the sulfonamide group and the nitrogen atom of the thiadiazol or thiazol rings were predicted to bind to the zinc ion as revealed by the molecular modelling studies. Dallavallea and co-worker reported phenyl-4-yl-acrylohydroxamic acid derivatives as potent HDACIs with cinnamic-based hydrazones and amino or hydroxyureas as ZBGs (**23**)^66^. In the activity studies, the derived molecules were discovered to be less effective than the parent hydroxamic acid derivatives. Wu and co-worker designed HDAC2 selective inhibitor β-hydroxymethyl chalcone (**24**), which exhibited about 20-fold isoform selectivity of HDAC2 (IC_50_ 0.17 μM) over HDAC1 (IC_50_ 2.74 μM)^67^. A reaction-mechanism-based pattern was proposed in the binding of the derived inhibitor to zinc ion by intramolecular cyclisation which was catalysed by the zinc atom.

Selectivity is considered to play important role in the safety and potency of HDACIs. The novel ZBGs not only functioned as zinc chelators in the structures of HDACIs, but also can influence the selectivity of the enclosed molecules. The imidazole thione and pyrimido[1,2-c][1,3]benzothiazin-6-imine derivatives exhibited inhibitory selectivity of HDAC8, which is implicated in cancer, schistosomiasis, and Cornelia de Lange syndrome^68^. The tropolone derivatives can selectively inhibit the bioactivity of both HDAC2 and HDAC8. The primary amide derivatives showed inhibitory selectivity of HDAC1, and the 3-HPT derivatives exhibited selectivity of HDAC6 over HDAC1. The chelidamic and hydrazide containing HDACIs featured selectivity of HDAC1, 4, and HDAC3, respectively. Two chemotypes of HDACIs even exhibited group-specific inhibitory selectivity of HDACs, such as the TFMO (with selectivity of class IIa HDACs) and 2-(oxazol-2-yl)phenol (with selectivity of HDAC1, 6, and 10) derivatives. The mentioned ZBGs can be applied to the design of HDACIs for the treatment of diseases or mechanistic studies by targeting a specific HDAC isoform or a specific group of HDACs.

## Conclusion and discussion

Inhibition of HDACs has achieved rapid development in recent years, and HDACIs have exhibited enormous therapeutic potential in the treatment of cancer and other diseases. More than 15 HDACIs have been investigated in clinical trials, and four of them have gained approval from the US FDA. As targeted antitumor drugs, the efficacy and safety of HDACIs in tumour treatment had been confirmed^69^. Therefore, development of HDACIs has attracted extreme attentions in the field of drug discovery. However, current development efforts focused on structural reorganisation and modification in the cap and linker motifs, while the ZBGs did not receive much attention, the classical ZBGs such as hydroxamic acid and benzamide groups were usually directly adopted into the new compounds.

Hydroxamic acid group is widely used as ZBGs due to its high zinc chelating ability, and benzamide is chosen for its class I HDACs selectivity. The application of carboxylic acid and thiol groups were limited by the lack of potency and only found in a small group of HDACIs. The potency and safety of HDACIs with the above ZBGs have been evaluated by various studies including different stages of clinical trials. Discovery of HDACIs with classic ZBGs, and especially the hydroxamic acid and benzamide derivatives, plays significant roles in the drug development by the inhibition of HDACs. Substituting the hydroxamic acid and benzamide groups with other groups usually resulted in reduction of activities comparing to the current highly active HDACIs. Moreover, the HDACIs designed with novel ZBGs from the beginning also have difficulties to show improved activities than those with the classical hydroxamic acid group.

In spite of the difficulties mentioned above, introduction of novel ZBGs is still necessary for the development of HDACIs of new chemotypes so that the pharmacokinetic and safety issues of current agents could be improved. Significant achievements have been gained in the development of HDACIs with novel ZBGs. Many have exhibited superior selectivity, such as the tropolone, 3-HPT, and TFMO derivatives. Some molecules also displayed surprisingly high potency in the activity assay, such as the hydrazide HDACIs discovered by Chou and co-worker (exhibited enzyme inhibitory activity with IC_50_ in the pM range and antiproliferative activity with IC_50_ in the nM range). Although none of the HDACIs with novel ZBGs has been approved by FDA or even gained access to the clinical trials, the future of these HDACIs as new generations is promising. The encouraging outcomes of these investigations also guaranteed further explorations on new ZBGs in HDACI development. Wide application of novel ZBGs in the HDACIs design will contribute to the emergence of new HDACIs.

### Perspectives

Zinc binding groups play significant role in the potency of HDACIs. The wide application of current ZBGs is restricted by the poor pharmacokinetics or selectivity. Thus, it is necessary to develop new types of ZBGs for the design of HDACIs with improved druggability. In order to be selected in the design of HDACIs, the new types of ZBGs should exhibit advantages in potency, selectivity, pharmacokinetics, or safety compared with the classic ZBGs. However, it is still a challenge for the new designed ZBGs to show better performance than the classic ZBGs in the design of HDACIs. Therefore, a vast amount of further research work is needed in the discovery of novel ZBGs with exploitable value. Structural derivatisation of current ZBGs, such as hydroxamic acid and benzamide groups, is believed to be an effective way of rapid discovery of ZBGs with development potential. Novel ZBGs can be efficiently derived by computing methods, reference to or modification of natural structures, screening of molecular data base, isostere substitution, and *de novo* design. Study on the metabolic patterns of current ZBGs also provides valuable information for the design of new ZBGs.
